# Supplementary material for: Towards a further understanding of measles vaccine hesitancy in Khartoum state, Sudan: A qualitative study
Source: PLoS One. 2019 Jun 20;14(6):e0213882. doi: 10.1371/journal.pone.0213882 (PMC6586394; doi:10.1371/journal.pone.0213882)
Supplement: S1 File — (DOCX) [file pone.0213882.s001.docx]

**Questions guide**

| 1. **Gender:**  1/ Male 2/ Female 2. **What is your age?**  ……………………………………. 3. **What is your academic qualification?**   …………………………………….   1. **Professional experience in vaccination provision (Years)?**   …………………………………….   1. **What do you think are the main issues or challenges faced by immunization programs at the moment in Sudan?** 2. **What do you think are the main reasons behind the low coverage of measles vaccine when compared with the other vaccines coverage? (especially the second dose of measles vaccine)?**  - What the effect of availability of measles vaccine in limited days during the week (two days per week)?  1. **Have you ever received any report about there are some people having doubts and concerns regarding measles vaccines? (Yes / No)**  - If yes, what are those doubts and concerns? - If yes, are people having doubts and concerns about the measles vaccine clustered in specific geographic area or subgroups (e.g. ethnic minority, etc.) - Do you think that doubts and concerns affected measles vaccine coverage in these clusters? To what extent?  1. **How would you define vaccine hesitancy?** 2. **Do you think measles vaccine hesitancy exists in Sudan? Why?** 3. **If yes, what do you consider to be the causes/ determinants of measles vaccine hesitancy in Sudan/Khartoum state?**  - **What is the role of Contextual influences?** (*Media/influential leaders, pro or anti-vaccination lobbies/ Religion, culture, Socio-economics/ Politics/ Geographic barriers)* - **What is the role of Individual and Groups influences?** *(Experiences with vaccination/ Beliefs, attitudes about prevention and health/ Knowledge, awareness/ Trust in health systems and providers/ Perceived risk vs benefit/ Immunization as a social norm vs not needed/harmful)* - **What is the role of Vaccine/ vaccination specific issues?** (*Risk vs benefit (scientific)/ Mode of administration/ Mode of delivery (time and availability)/ Schedule/ The strength of recommendation, knowledge base or attitude of vaccine providers).*  1. **Is measles vaccine hesitancy focused in any specific geographic areas in Sudan/Khartoum state?** (Yes / No)  - If yes, where do you consider to be these/ this geographic area(s)? What are their reasons behind measles vaccine hesitancy?  1. **Is measles vaccine hesitancy focused on specific groups of people (Ethnic, religious or sociocultural/ socioeconomic) in Sudan/ Khartoum state?** 2. **Do you think measles vaccine hesitancy has any impact on the measles vaccination program in Sudan? (Yes/ No)**  - If yes, How, and to what extent? - If no, Why not? |
| --- |
